# Hidradenitis Suppurativa Recurrence in a Caesarean Scar

**DOI:** 10.1155/2020/6283720

**Published:** 2020-06-03

**Authors:** K. M. Darch, T. L. Holland, L. J. Spelman

**Affiliations:** Veracity Clinical Research, Suite 18 Level 1 250 Ipswich Rd., Woolloongabba QLD 4102, Australia

## Abstract

Hidradenitis suppurativa (HS), also known as acne inversa, is a chronic, relapsing inflammatory skin condition characterised by the presence of painful nodules, abscesses, and sinus tracts or scarring. Affecting up to 4% of the population, it is not uncommon and is seen predominantly in females at a ratio of 3 : 1. HS carries a substantial burden for those who suffer from it, from the significant psychosocial impact, to the cost of the multitude of topical and systemic treatments which often do not successfully control its symptoms. In this case report, we discuss a 33-year-old female known to our clinic, who presented with a recurrence of her HS in a caesarean scar, with otherwise silent disease. From our review of the literature, this appears to be only the second case of recurrence of HS in a caesarean scar reported to date. With a predilection for females of reproductive ages, involvement of sensitive areas, and an average of greater than seven years from onset of symptoms until diagnosis, the ability to recognise HS and ensure referral for specialist management is essential for all who are regularly involved in the management of this patient group.

## 1. Introduction

Affecting up to 4% of the population, HS is a common, chronic and debilitating disease, seen more commonly in females with a ratio of 3 : 1 [1]. It may occur at any time from the age of puberty, with only 2% of cases occurring before the age of 11 [2]. The exact aetiology of HS and its resultant painful nodules, abscesses, sinus tracts, and scarring remain unknown; however, recent evidence points to the dysregulation of skin immunity around hair follicles in affected regions [1]. Its sequelae, painful nodules, abscesses, and sinus tracts, are thought to be the result of inflammation of the hair follicle and resultant hyperkeratosis which leads to follicular occlusion and subsequent rupture of the follicle, with the release of the contents of the follicle perpetuating the inflammatory response of the surrounding dermis [1]. Studies have revealed elevated levels of IL-1b, TNF, IL-10, and IL-17 in the lesional skin of those with HS and have shown abnormalities in antimicrobial peptides and Toll-like-receptor signalling [1, 3].

The longstanding theory that HS is the result of infection of the apocrine sweat glands is no longer supported, with studies revealing that cultures taken from discharging lesions of HS most commonly show either no growth or growth of normal skin flora [1], though the secondary infection of sinus tracts and abscesses is possible. HS is known to beassociated with a number of other conditions including inflammatory bowel disease, depression and other psychiatric conditions, and the metabolic syndrome [1], highlighting the importance of a multidisciplinary approach to its management.

## 2. Case

This is the case of a 33-year-old female known to our clinic for management of HS, successfully managed with Humira (adalimumab) 40 mg injections weekly. She became pregnant with her first child and continued Humira until the end of the second trimester. She gave birth via emergency caesarean at 39 weeks to a healthy 3.59 kg male following induction of labor, failure to progress, and foetal bradycardias. Her HS remained silent throughout the pregnancy and after the birth, until approximately one week later when three painful nodules appeared in her left axilla. She self-recommenced Humira at this time, after ceasing breast feeding, and these nodules settled. Approximately two months later, she noted the appearance of small nodules and inflammatory tracts around her caesarean scar. These were initially thought to represent a fungal infection and were treated with an over-the-counter antifungal cream with limited efficacy. She was subsequently treated with topical antimicrobial preparation containing gramicidin, neomycin sulfate, nystatin, and triamcinolone acetonide, followed by oral amoxycillin and clavulanic acid, and potassium permanganate soaks to the area, all of which provided minimal improvement. Our patient reported excellent compliance with all wound care instructions at the time of delivery and noted significant distress at being told that her symptoms were the result of insufficient wound care and recurrent infection of the area.

She was seen in our clinic one month after the onset of symptoms. We noted the presence of a number of small, well-defined HS lesions, with nodules and discharging sinus tracts around the caesarean scar. Her disease elsewhere remained silent, and her compliance with Humira was excellent. She was subsequently diagnosed with a recurrence of HS in her caesarean scar, providing her with a sense of relief following the multiple previous presentations and reviews.

Her HS lesions involving her caesarean site responded well to treatment with doxycycline 100 mg daily and Prontosan Wound Gel and Wash (polyaminopropyl biguanide (polihexanide), betaine surfactant) in addition to Humira, with discharging sinus tracts persisting until review six months after the onset of symptoms, and complete resolution by review at one year.

## 3. Discussion

Our extensive search of the literature reveals only one other case of a recurrence of HS in a caesarean scar. This recently published report describes the case of a 34-year-old female with Hurley Stage II HS since the age of 18. The appearance of nodules and discharging fistulae were noted approximately five months following her second caesarean section, with the appearance of similar nodules also noted following her first caesarean section. Unlike our patient, she was also noted to have active disease in bilateral axillae. She was treated with doxycycline 100 mg twice a day, and topical treatment with both the antiseptic octenidine and a clindamycin/benzoyl peroxide gel [4]. There has been one other case report published which describes wound dehiscence following caesarean section in a female with HS, with multiple other comorbidities including obesity, gestational diabetes, and current smoker. The case report described that wound dehiscence was common following excision of HS lesions and as a result suggested that HS was the cause for the patient's postoperative complications [5].

The potential for HS to show Koebnerisation, or the predilection for areas of injury or trauma to otherwise “normal” skin, has been discussed in the literature previously. A case series that detailed 14 patients with HS lesions in locations not typical for the disease has described the presence of lesions, including nodules, abscesses, cysts, and in one patient a draining sunus tract, at the level of the waistband [6]. Seven of these patients had lesions excised, with histopathology results supporting the diagnosis of HS in all cases. All of the participants were obese, and all had a history of HS elsewhere. From this, the authors hypothesised that these HS lesions were the result of Koebnerisation following “traumatisation from external factors.” They provide the details of another two studies, both describing female participants who were also obese and had a history of HS, in whom HS lesions were found to be present in line with where the edge of their bra sits [7, 8] and suggest that this may also be the result of Koebnerisation.

Further to this, Boer et al. investigated the potential for mechanical stressors to cause Koebnerisation of HS [9]. They suggested that the biomechanical stressors on inverse and concave skin areas are different to that in other areas, and that these constant mechanical forces are enhanced in obese patients and could be the reason for HS's predilection for these areas. They also note that mechanical stress on the skin induces a proinflammatory environment, epidermal hyperplasia, and hyperkeratosis and cited other associated conditions such as acne mechanica, which develops in areas exposed to mechanical stressors.

### 3.1. Management of HS

Successful management of HS remains a significant clinical challenge. The average time from onset of HS symptoms to diagnosis is 7.2 years [10]. This delay perpetuates the distress experienced by those who suffer from HS and is thought to result from of a lack of familiarity of clinicians with the condition, the frequent misdiagnosis of the inflammatory lesions of HS as recurrent boils or folliculitis which understandably fail to respond to conventional antimicrobial therapies [11] and the resultant reluctance of patients to continue to present to healthcare providers. Education, advocacy, referral, and early diagnosis are essential for adequate management of this population of patients. It has been suggested that in conjunction with medical therapies, all patients must receive adequate counselling and support for known comorbidities, including adequate analgesia, support to lose weight and for smoking cessation, treatment of superinfection, and proper education regarding management of active lesions [2].

Given the population most commonly affected by HS, knowledge of best management of the disease in women who are considering pregnancy, who are pregnant, and who are breast feeding is essential. Most women experience no change in their HS during pregnancy, with 20% reporting improvement while pregnant, and 8% experiencing worsening of their disease [12]. An article by Perng et al. [12] published in 2017 provides a summary of the treatment options available for women during pregnancy. They discuss the use of topical antibiotics, with clindamycin (1%), metronidazole (0.75%), and erythromycin (2%) classified as FDA pregnancy category B drugs. Though often considered to be first-line treatment in the non-pregnant population, the use of oral tetracyclines is contraindicated during pregnancy, and they are not recommended for use in lactation. The combination of oral clindamycin and rifampicin may provide significant benefit for patients with moderate-to-severe HS, and with clindamycin listed as pregnancy category B, and rifampicin as pregnancy category C, a short course of this combination therapy may be considered in pregnant patients where the benefit of treatment outweighs any potential risks, and where there are no other contraindications to their use or drug-drug interactions.

Biologic agents such as adalimumab and infliximab are considered to be category B drugs in pregnancy; however, the evidence to support their use in pregnancy is controversial [12, 13]. Given the high molecular weight of adalimumab, it is likely to require active transport to cross the placenta [14]. For this reason, it is suggested that biologic agents such as adalimumab should be ceased prior to the third trimester of pregnancy when active transport of maternal IgG occurs in order to limit potential foetal exposure [12, 14]. Evidence for their use in breastfeeding is also limited.

### 3.2. Psychological Impact of HS

HS has been said to cause “tremendous psychological stress” for those who suffer from it [15]. The psychosocial impact of HS may be mediated by a number of factors. The pain and discharge from draining sinuses and abscesses may significantly impair a patient's ability to work, exercise, and socialise. The physical appearance of the lesions and their predilection for sensitive areas often cause significant shame and embarrassment for those who suffer from them, which again has an impact on their ability to work and socialise and on their relationships. HS remains a significant challenge to successfully manage, and the expense of multiple topical and systemic treatments also carries a significant impact.

Studies have shown that depression is diagnosed at a higher rate (5.9%) in those with HS when compared to the rate in matched controls (3.5%), and at a higher rate in females who suffer from the condition [12]. “Anxiety” was similarly diagnosed at a higher rate in those with HS (3.9%) when compared to matched controls (2.4%) [12], and the study discussed a nonstatistically significant increase in the rate of diagnosis of other psychiatric conditions such as schizophrenia and bipolar disorder in those who suffered from HS compared to matched controls.

For our patient, the fact that she felt that she was not listened to and that she was told it was not possible for her HS to recur in a caesarean scar added significantly to her distress.

## 4. Conclusions

In this case report, we have discussed the recurrence of HS in a caesarean scar of a patient with otherwise silent disease. This appears to be only the second case of this that has been reported in the literature to date, with a similar case recently published. We have discussed the significant burden that HS and misdiagnosis of the condition have on those who suffer from it. Patient education, early referral, and a multidisciplinary team approach to management may greatly assist to ease this disease burden and may have significantly improved the experience of our patient.

Given that HS is more likely to affect women and can be seen from the age of puberty, a knowledge of the management of HS during pregnancy and breast feeding forms an essential part of the holistic management of the HS patient. We hope that this case may serve as a reminder of the significant distress that HS can cause, that it may present in atypical sites, and the difference that holistic management can make.
